# Cost of pre-exposure prophylaxis delivery in family planning clinics to prevent HIV acquisition among adolescent girls and young women in Kisumu, Kenya

**DOI:** 10.1371/journal.pone.0249625

**Published:** 2021-04-15

**Authors:** Valentine Wanga, Kathryn Peebles, Alfred Obiero, Felix Mogaka, Victor Omollo, Josephine B. Odoyo, Jennifer F. Morton, Elizabeth A. Bukusi, Connie Celum, Jared M. Baeten, Ruanne V. Barnabas

**Affiliations:** 1 Department of Epidemiology, University of Washington, Seattle, WA, United States of America; 2 Department of Global Health, University of Washington, Seattle, WA, United States of America; 3 Centre for Microbiology Research, Kenya Medical Research Institute, Nairobi, Kenya; 4 Departments of Obstetrics and Gynecology, University of Washington, Seattle, WA, United States of America; 5 Department of Medicine, University of Washington, Seattle, WA, United States of America; Boston University School of Public Health, UNITED STATES

## Abstract

**Introduction:**

Oral pre-exposure prophylaxis (PrEP) is increasingly being implemented in sub-Saharan Africa. Adolescent girls and young women (AGYW) in Kenya contribute more than half of all new infections among young people aged 15–24 years, highlighting the need for evidence on the cost of PrEP in real-world implementation to inform the budget impact, cost-effectiveness, and financial sustainability of PrEP programs.

**Methods:**

We estimated the cost of delivering PrEP to AGYW enrolled in a PrEP implementation study in two family planning clinics in Kisumu county, located in western Kenya. We derived total annual costs and the average cost per client-month of PrEP by input type (variable or fixed) and visit type (initiation or follow-up). We estimated all costs as implemented in the study, and under implementation by the Kenyan Ministry of Health (MoH), both at the program volume observed and if the facilities were delivering PrEP at full capacity (scaled-MoH).

**Results:**

For the costing period between March 2018 and March 2019, 615 HIV-negative women contributed 1,128 (502 initiation and 626 follow-up) visits. The average cost per client-month of PrEP dispensed per study protocol and per the MoH scenario was $28.92 and $14.52, respectively. If the MoH scaled the program so that facilities could see PrEP clients at capacity, the average cost per client-month of PrEP was $10.88. Medication costs accounted for the largest proportion of the total annual costs (48% in MoH scenario and 65% in the scaled-MoH scenario).

**Conclusions:**

Using data from a PrEP implementation program, we found that the cost per client-month of PrEP dispensed is reduced by 62% if PrEP delivery at the two clinics is scaled up by the MoH. Our findings are valuable for informing local resource allocation and budgetary cost projections for scale-up of PrEP delivery to AGYW. Additionally, previous cost-effectiveness studies have been limited by the use of fixed assumptions of the cost of PrEP per person-month. Our study provides cost estimates from practical data which will better inform cost-effectiveness and budget impact analyses.

## Introduction

In 2015, the World Health Organization (WHO) expanded its recommendation on the use antiretroviral therapy as pre-exposure prophylaxis (PrEP) for the prevention of HIV transmission [1]. Since then, 35 countries have issued guidelines indicating PrEP as an additional HIV combination prevention to priority populations including adolescent girls and young women (AGYW) aged 15–24 years, who account for a disproportionate number of new infections [2]. In Kenya, AGYW contribute more than half of all new HIV infections among young people aged 15–24 years [9], underscoring the need to identify the cost of delivering PrEP to AGYW in order to inform the most cost-effective ways of PrEP delivery in the country.

Health facilities are the most common platforms for PrEP delivery for AGYW in sub-Saharan Africa [3]. These platforms offer the benefit of reaching individuals who are using other services such as HIV testing and family planning, but the cost of PrEP delivery in these settings remains unknown. Moreover, low- and middle-income countries (LMICs) still rely on donor funding for their HIV response, with an estimated 44% of the total resources for HIV in LMIC reported to have come from external sources in 2018 [4]. In the context of limited funding for HIV response, uncertainty about PrEP delivery costs leaves decision-makers unable to determine if PrEP is affordable or cost-effective within a given budget. Cost estimates from PrEP implementation projects are needed to provide actionable insights on the financial implications of PrEP delivery to at-risk populations.

Evidence from mathematical modelling of PrEP in sub-Saharan Africa suggest that compared to no PrEP, PrEP can be a cost-saving intervention for AGYW if implemented in combination with other HIV prevention strategies [5–7]. However, these models had fixed assumptions about the cost of PrEP per person-year and did not use data from real-world implementation settings. To date, only one study has reported the cost of delivering PrEP to AGYW in sub-Saharan Africa using practical data from implementation [8], and more evidence is needed on the cost of delivering PrEP to this population.

The objective of this study was to evaluate the cost of delivering daily oral PrEP to AGYW in two family planning clinics in Kisumu, one of the three counties in western Kenya with a 2018 HIV incidence of >2.61 per 1000 and prevalence >11.1% [9]. We estimated the total annual cost and average cost per client-month of PrEP dispensed (1) as implemented in the study setting and (2) as would be incurred by the Kenyan Ministry of Health (MoH) if it were to implement PrEP delivery to the same population in the same facilities.

## Methods

### Study setting and population

Prevention Options for Women Evaluation Research (POWER) is an ongoing implementation science study evaluating PrEP delivery to AGYW aged 16–25 years in Kenya and South Africa [3]. This costing study was conducted for the two POWER study sites in Kisumu, Kenya (Jaramogi Oginga Odinga Teaching and Referral Hospital (JOOTRH), a public facility and Kisumu Medical Education Trust (KMET), a youth-friendly private facility). Study enrollment started in August 2017 at KMET and in October 2017 at JOOTRH. AGYW were eligible for enrollment in the study if they were 16–25 years old, able and willing to provide written informed consent, recently sexually active (defined as having had vaginal intercourse at least once in the previous three months) and HIV-uninfected at enrollment. Scheduled follow-up visits in the study were one month post-enrollment, three months post-enrollment and quarterly thereafter, per the Kenyan national guidelines [10]. The POWER study protocol was reviewed and approved by the Human Subjects Division of the University of Washington and the Scientific and Ethics Review Unit of the Kenya Medical Research Institute. The study included women of ages 16–25, and we followed local guidelines for consent for those under 18 years of age. All participants provided written informed consent to participate in the study.

### Data collection

We collected PrEP delivery costs of programmatic activities including 1) direct service delivery activities: counseling, HIV testing, laboratory testing (creatinine clearance, hepatitis B surface antigen, *Neisseria gonorrhoeae* (NG) and *Chlamydia trachomatis* (CT)), PrEP prescribing and dispensing; 2) ancillary activities: demand creation (flyers, posters, t-shirts, and quarterly support groups for POWER clients) and adherence support through appointment reminder calls; and 3) site-level activities: initial PrEP training, annual refresher trainings, and supervision and administration (monthly reporting of PrEP uptake and continuation, monthly PrEP accounting). Additionally, we collected costs of capital inputs (vehicle, clinical equipment, stationery) and overhead (building, utilities, fuel, maintenance, internet).

We obtained personnel salaries, startup costs (e.g., training, capital), and recurrent costs (e.g., clinical supplies, rent, utilities) from study salary records, expense reports and receipts. We obtained facility staff and ministry of health (MoH) salaries from communication with site staff and publicly-available information on job groups and basic allowances (housing and commuter) per job cadre [11]. Public sector cost per bottle of tenofovir disoproxil fumarate/emtricitabine (TDF/FTC, $6.25 in 2017 US dollars) was obtained from recently published data from Kenya [8]. We conducted time-and-motion observations to record the time spent by providers during each direct PrEP delivery activity. Through interviews with study personnel, we also collected data on the proportion of time spent on ancillary PrEP activities by each staff. Study data were used to determine the number of initiation and follow-up visits and the number of PrEP months dispensed in the one-year costing period. We collected all costs in Kenyan shillings (KES) and converted them to U.S. dollars at the market exchange rate (1 USD = 100.7 KES) on August 30, 2018, the mid-point of the costing period.

### Cost analysis

Using the Global Health Cost Consortium Reference Case (GHCC) principles [12], we evaluated the economic costs associated with PrEP services from a provider perspective for the period between March 1, 2018 and March 1, 2019. We used methods similar to Roberts et.al. [8], to derive total annual costs and average cost per client-month of PrEP (defined as total annual cost divided by the months of PrEP dispensed in a year) by input type (variable or fixed) and visit type (initiation or follow-up). All costs were estimated 1) as incurred by the study (POWER study scenario); 2) as would be incurred by the MoH if it were to implement the program (MoH scenario) and 3) as would be incurred by the MoH if the program were to be scaled at the two clinics (scaled-MoH scenario). We estimated the total number of daily visits in the scaled-MoH scenario as the weighted average of the total time of initiation and follow-up visits, with weights assigned as the proportion of visits of each type observed in the costing period–the final cost estimates took into account the labor costs of all staff involved in an initiation or follow-up visit.

Clinical personnel, medication (TDF/FTC) and laboratory testing were categorized as variable inputs, while fixed inputs included training (start-up and refresher), demand creation, personnel (supervision and administration), capital and overhead. To calculate personnel costs, we used total salary per staff (including allowances) and assumed each staff worked eight hours per day for 260 week days per year after excluding standard holidays and leave/vacation. We used the average time clinical staff spent on PrEP delivery activities to estimate unit labor costs per activity. To estimate fixed costs by visit type, we calculated the proportion of total average visit time contributed by each visit type, and multiplied each proportion by the total cost of fixed inputs.

We added the shipping cost per bottle of PrEP ($2.18) to the public sector cost per bottle ($6.25) to estimate a drug cost of $8.43 per bottle for the program; costs of drug storage were included in building and utility costs. For MoH and scaled-MoH scenarios, we added 8% central storage and distribution fees per Roberts et.al. [8], to the public sector cost per bottle for a total cost of $6.75 per bottle (Table 1). Total medication costs were determined by multiplying the unit price by the number of months of PrEP dispensed during the costing period. The total cost of laboratory and HIV testing was estimated as the unit cost of tests and clinical supplies multiplied by the number of tests done during the costing period.

**Table 1:** Differences inputs per scenario.

	POWER study scenario	MoH and scaled MOH scenarios*
Variable inputs		
Personnel (clinical staff)	POWER study salaries	MoH Salaries and allowances
Medication	$8.43 per bottle; 1554 months of PrEP dispensed	$6.75 per bottle; months of PrEP dispensed (1554 in MoH scenario, 7645 in scaled-MoH scenario)*
Laboratory and HIV testing	Unit cost of $58 per dual NG/CT GeneXpert test cartridge	Unit cost of $16 per dual NG/CT GeneXpert test cartridge
Fixed inputs		
Training (start-up)	2-day training	1-day training
Training (refresher)	Conducted annually	Weekly, hour-long CMEs with PrEP once a month
Demand creation	T-shirts, flyers, posters, POWER Queens meetings (support groups)	Only flyers and posters and support groups
Personnel (supervision and administration)	POWER study salaries	MoH Salaries and allowances
Capital (vehicle, furniture, clinical equipment)	Vehicle purchased; 7 laptops; creatinine machine (unit cost of KES 450,000) used at JOOTRH	No vehicle; 2 laptops; StatSensor Xpress creatinine machine (unit cost of KES 10000) used at JOOTRH
Overhead (building, utilities, fuel, maintenance, internet)	Fuel, maintenance and insurance included; two rooms per facility	No fuel and vehicle maintenance; one room per facility

MoH = Ministry of Health; PrEP = Pre-exposure prophylaxis; NG = Neisseria gonorrhoeae, CT = Chlamydia trachomatis; CME = continuous medical education; JOOTRH = Jaramogi Oginga Odinga Teaching Referral Hospital.

*The only difference between MoH and scaled MoH scenarios is the program volume, i.e., the months of PrEP dispensed.

For most inputs, we used POWER study costs to derive MoH and scaled-MoH costs. Key differences in the MoH scenarios, per consultation with site staff, were: 1) we used MoH salaries and allowances for personnel costs; 2) start-up training would only take one day instead of two days; 3) annual POWER refresher trainings would be similar to weekly, hour-long continuous medical education (CMEs) and that PrEP would be discussed at CMEs once a month; 4) demand creation would only be done through flyers and posters; 5) no vehicle would be purchased since costs of drug transportation (what the vehicle was mainly used for in the study) are already included in the drug price; thus, there would be no fuel, maintenance and insurance costs; 6) only one laptop per facility would be purchased for PrEP use at the health records department; 7) at JOOTRH, StatSensor Xpress [8] creatinine machine (unit cost of KES 10000) would be purchased instead of the machine used for the study (unit cost of KES 450,000); 8) only one room per facility would be used for PrEP activities; and 9) for sexually-transmitted infection (STI) testing costs, we used a unit cost of $16 per dual NG/CT GeneXpert test cartridge (T. Elvira, personal communication), and assumed, given the number of people living with HIV (1.5 million [9]), relative to the number of PrEP enrollees in Kenya (55,500–56,500 [2]), that the cost of GeneXpert machine use (if already procured for TB and HIV viral load testing) attributable to PrEP would be negligible (Table 1).

Per POWER study protocol, all study participants were tested for HIV at each visit, and for creatinine clearance (CrCl), Hepatitis B surface antigen (HBsAg) and STIs (NG and CT) at PrEP initiation. Per the national PrEP guidelines in Kenya, testing for CrCl, HBsAg and STIs is recommended but not required at PrEP initiation [10]. Furthermore, in Kenya, as in almost all parts of Africa, etiologic STI testing using nucleic acid amplification is not routinely done in facilities, and STI diagnoses and treatment are made using syndromic assessment [13]. Therefore, in our primary analyses, we estimated explored costs without CrCl, HBsAg or STI testing. For secondary analyses, we explored the incremental cost of 1) CrCl, HBsAg and STI testing; 2) MoH-recommended CrCl and HBsAg testing but no STI testing and 3) only STI testing, since STI prevalence is considerably higher in this population [14–16] than kidney issues revealed through CrCl [8, 17].

We excluded all research-related costs and visits by clients who never initiated PrEP (7.5% of all visits). Start-up and capital costs were annualized using a discount rate of 3% [12] over the expected useful life of three (e.g., t-shirts) to five (e.g., vehicle) years. All cost estimates are reported in 2019 USD with adjustment for inflation from 2017 [18], the year POWER study was initiated. Program data extraction was done using SAS version 9.4 (SAS Institute Inc., Cary, NC, USA), and cost data analysis was done using Excel 2016 (Microsoft, Redmond, WA, USA). The excel file used for analysis is available in the (S1 File).

## Results

### Program volume summary

Between March 2018 and March 2019, 615 HIV-negative women (310 at JOOTRH and 305 at KMET) contributed 1,128 (502 initiation and 626 follow-up) visits, with a range of one to six visits per woman. PrEP was dispensed at 75% (N = 471) of all follow-up visits in this period. There were 1,554 (499 at initiation visits, 995 at follow-up visits) months of PrEP dispensed during the one-year period. We conducted 36 time-and-motion observations (13 initiation and 23 follow-up visits), and on average, initiation visits and follow-up visits took 50 minutes and 24 minutes of providers (nurses, HIV testing counselors, laboratory technicians and pharmacy technicians) time, respectively.

Of the visits included in the one-year costing period, 44.5% were initiation visits (all with PrEP dispensed), 19.8% were month one (PrEP dispensed in 82%) visits and 35.7% were quarterly (PrEP dispensed in 72%) visits. Based on the total time providers worked per day at each facility (450 minutes), the proportion of initiation and follow-up visits seen in the costing period and the average time per initiation and follow-up visit from time-and-motion data, we estimated that at full capacity, 14 PrEP visits could be seen per day per facility (i.e., initiation visits per day = 0.45 × 450/50 = 4, follow-up visits per day = 0.55 × 450/24 = 10). For 260 work days a year, we estimated that 7,280 (3,240 initiation and 4,040 follow-up) visits would occur in the scaled-MoH scenario in a year, during which 7,645 months of PrEP would be dispensed (Table 1). Details of how these numbers were obtained are in the “Program Volume” sheet of the S1 File.

### PrEP delivery costs (without creatinine, hepatitis B or STI testing)

**Fig 1:**
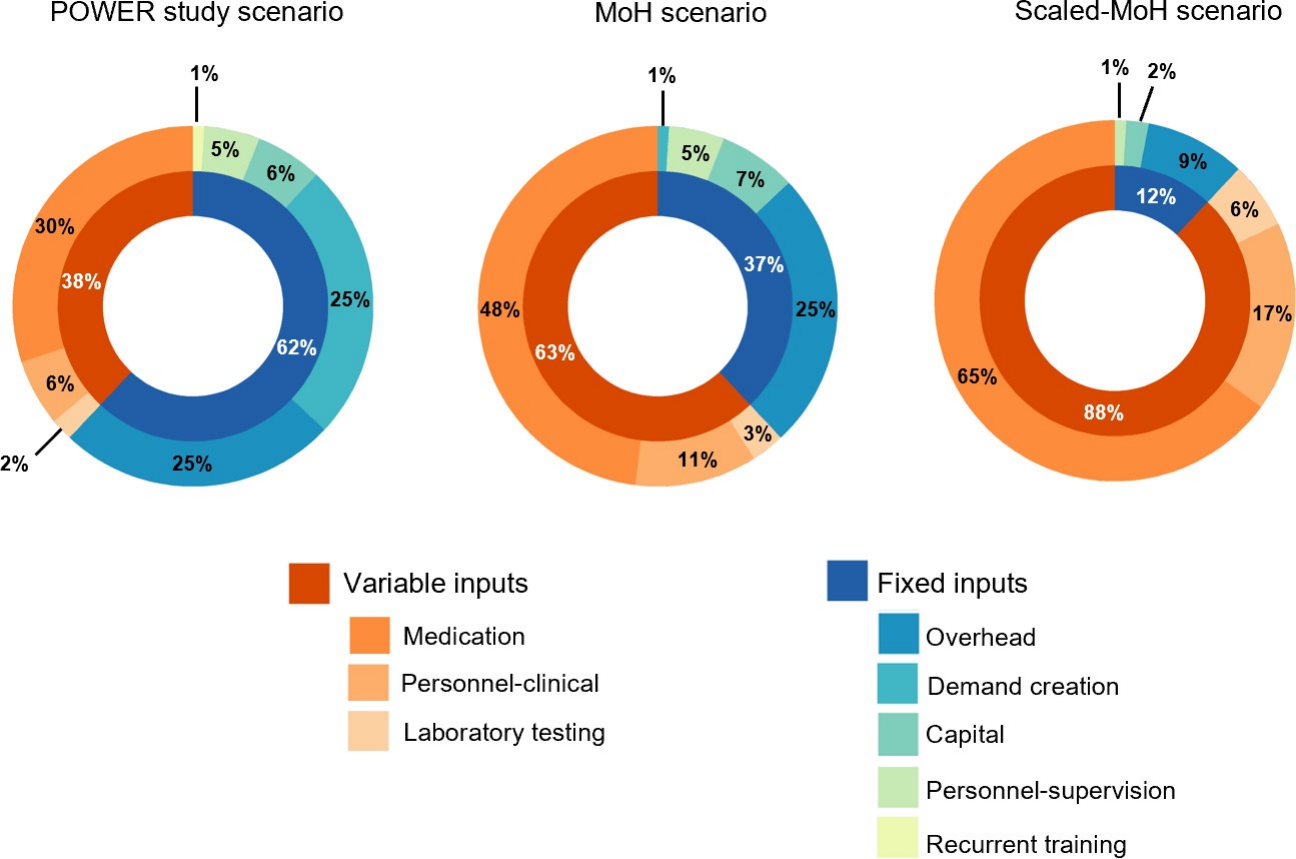
Proportion of total annual cost (2019 USD) by input type. Only proportions >0.5% are presented. In the Ministry of Health scenario, recurrent training contributed 0.02%. In the Scaled Ministry of Health scenario, demand creation and recurrent training contributed 0.15% and 0.01%, respectively.

In the MoH scenario, (if MoH implemented the program), the total annual cost of PrEP delivery was $22,566 and the cost per client-month of PrEP dispensed was $14.42, representing half the POWER study costs (Table 2); medication was the main driver of the total annual cost (48%), followed by overhead costs (25%), while demand creation contributed only a small proportion (1%) to total annual costs (Fig 1). In the scaled-MoH scenario, the cost per client-month of PrEP dispensed was $10.88, with medication, personnel and overhead contributing 65%, 19% and 9% of total costs, respectively (Fig 1).

**Table 2:** Estimated total and average economic costs (2019 USD).

	POWER study scenario		MoH scenario		Scaled-MoH scenario	
	Annual cost	Cost/client-month of PrEP	Annual cost	Cost/client-month of PrEP	Annual cost	Cost/client-month of PrEP
Variable inputs						
Personnel (clinical staff)	2705	1.74	2562	1.65	14536	1.90
Medication	13620	8.77	10907	7.02	53670	7.02
Laboratory and HIV testing	782	0.50	782	0.50	5044	0.66
Variable total	17107	11.01	14251	9.17	73250	9.58
Fixed inputs						
Training (start-up)	97	0.06	52	0.03	52	0.01
Training (refresher)	257	0.17	6	0.00	6	0.00
Demand creation	11436	7.36	125	0.08	125	0.02
Personnel (supervision and administration)	2391	1.54	1089	0.70	1089	0.14
Capital (vehicle, furniture, clinical equipment)	2513	1.62	1478	0.95	1478	0.19
Overhead (building, utilities, fuel, maintenance, internet)	11133	7.16	5566	3.58	7198	0.94
Fixed Total	27826	17.91	8315	5.35	9946	1.30
Total variable and fixed costs	44933	28.92	22566	14.52	83196	10.88

MoH = Ministry of Health; PrEP = Pre-exposure prophylaxis.

Assessing costs by visit type, we found that the total cost per initiation visit was $46.85 in the POWER study scenario, $20.23 in the MoH scenario and $11.84 in the scaled-MoH scenario. Follow-up visits cost less, at a total cost per visit of $33.38, $19.16 and $10.70 in the POWER study, MoH and scaled-MoH scenario, respectively. Compared to the total cost per initiation visit, the total cost per follow-up visit was 1.4 times higher in the POWER study, 1.1 times higher in the MOH and 1.1 times higher in the scaled-MoH scenarios (Table 3). The variable inputs associated with the decrease in costs of follow-up visits compared to initiation visits were personnel ($1.11 vs $3.20 per client-month of PrEP) and lab and HIV testing ($0.44 vs $0.70 per client-month of PrEP). Details of estimates by input type per visit type are in the S1 File (rows 23–50 of the “Total cost summary” sheet).

**Table 3:** Economic costs by visit type (2019 USD).

Visit type	POWER study scenario		MoH scenario		Scaled-MoH scenario	
	Annual cost	cost per client-month of PrEP	Annual cost	cost per client-month of PrEP	Annual cost	cost per client-month of PrEP
Initiation						
Variable costs	6327	12.67	5149	10.31	33346	10.29
Fixed costs	17194	34.42	5007	10.02	5007	1.55
Total variable and fixed	23520	47.09	10156	20.33	38352	11.84
Follow-up						
Variable costs	10264	10.31	8689	8.73	39905	9.06
Fixed costs	10633	10.68	3308	3.32	3308	0.75
Total variable and fixed costs	20896	20.99	11997	12.05	43213	9.81

MoH = Ministry of Health; PrEP = Pre-exposure prophylaxis.

### Added costs of creatinine, hepatitis B and STI testing

In a scenario with CrCl, HBsAg and STI testing at initiation, most of the total annual cost came from laboratory and HIV testing; 33% in MoH scenario and 47% in scaled-MoH scenario. Adding these three tests at initiation notably increased the cost per client-month of PrEP dispensed to $21.12 (45% increase) in the MoH scenario and $19.39 (78% increase) in the scaled-MoH scenario (Table 4).

**Table 4:** Economic costs by laboratory testing at initiation visit (2019 USD).

	POWER study scenario*		MoH scenario**		Scaled-MoH scenario**	
	Annual cost	Cost per client-month of PrEP	Annual cost	Cost per client-month of PrEP	Annual cost	Cost per client-month of PrEP
**CrCl, HBsAg and STI testing**						
Laboratory and HIV testing (% of total cost)	31936 (41%)	20.55	10830 (33%)	6.97	59911 (47%)	9.14
Total cost	77275	49.73	32810	21.12	148245	19.39
**CrCl and HBsAg testing only**						
Laboratory and HIV testing (% of total cost)	2477 (5%)	1.59	2477 (10%)	1.59	13702 (17%)	2.09
Total cost	47817	30.77	24457	15.74	94333	12.34
**STI testing only**						
Laboratory and HIV testing (% of total cost)	30240 (41%)	19.46	9135 (30%)	5.88	50533 (43%)	7.71
Total cost	74392	47.88	30919	19.90	137107	17.93

*Study scenario = costs if paid for by the POWER study

**MoH scenario = costs if paid for by MoH; CrCl = Creatinine clearance; HBsAg = Hepatitis B surface antigen; STI = *N*. *gonorrhoeae* and *C*. *trachomatis*; MoH = Ministry of Health; PrEP = Pre-exposure prophylaxis.

Excluding STI testing while performing CrCl and HBsAg testing at initiation resulted in minimal increases in total annual cost of PrEP delivery (8% in MoH scenario and 13% in scaled-MoH scenario). In comparison, when testing for STIs only, the average cost per client-month of PrEP dispensed was $19.90 (37% higher) and $17.93 (65% higher) in the MoH and scaled-MoH scenario, respectively (Table 4).

## Discussion

In this economic analysis among AGYW attending family planning clinics, we estimated the cost of Ministry of Health PrEP delivery in this setting to be $14.52 per client-month of PrEP dispensed. Under a scaled-MoH scenario, assuming 14 clients were seen at each facility per day, fixed costs would be distributed over a larger proportion of PrEP visits, significantly lowering the cost per client-month of PrEP to $10.88. Consistent with other costing studies of PrEP implementation [8, 19, 20], medication costs accounted for the majority of the total annual cost in both the MoH and scaled-MoH scenarios, highlighting the need to identify ways to further lower medication costs in PrEP delivery. On average, initiation visits took longer than follow-up visits (50 vs 24 minutes), increasing the personnel and laboratory and HIV testing costs of initiation visits. The proportion of time allocated to initiation vs follow-up visits visits (62% vs 38%) also explains the difference in fixed costs between follow-up and initiation visits ($10.68 vs $34.42 per client-month of PrEP).

At PrEP initiation, the Kenyan MoH recommends STI assessment (usually syndromic) and if available, laboratory evaluation of CrCl and HBsAg [10]. When we included costs of testing for CrCl, HBsAg and STIs (NG/CT (by nucleic acid amplification)) at initiation visits, laboratory costs contributed the majority of total annual costs and increased cost per client-month of PrEP by 45% in the MoH scenario (to $21.21) and 70% in the scaled-MoH scenario (to $19.39). Of the 502 women who initiated PrEP in the costing period, 7% and 17% tested positive for N. gonorrhoeae and C. trachomatis, respectively. Other studies have also shown high rates of NG and CT among young people, with even higher rates among women compared to men [21]. In PrEP users, high rates of STIs have been reported at baseline mostly among MSM populations [14] but also in young African women [22]. Because PrEP does not confer protection against STIs, and those at high risk of HIV are also usually at high risk of bacterial STIs which confers risk of infertility and other adverse reproductive health outcomes for women, there is a unique opportunity for PrEP programs to integrate STI testing and treatment with PrEP delivery. Still, the high unit cost of lab testing in this study ($58.53 per test in the POWER study and $16.00 per test in the MoH scenario) may not be sustainable in resource-limited settings. Furthermore, existing STI testing systems are inefficient due to long waiting times for laboratory results and the requirement that patients return to the clinic for treatment. Reliable, low-cost point-of-care testing for STIs are needed to address the high burden of STIs in young women in sub-Saharan Africa [15, 16].

Although we assumed that demand creation would happen only through posters and flyers in the MoH scenarios, research findings suggest that stigma remains a barrier to PrEP implementation at individual, community and provider levels [23, 24]. In the POWER cohort in Kisumu, additional demand creation was done through giving out t-shirts and having quarterly support groups with PrEP educational sessions for the women; with these additional components, demand creation contributed to 26% of total annual costs. Though we were unable to quantify the direct impact of these demand creation activities, study staff reported increased knowledge and awareness about PrEP among study participants and their friends who accompanied them to the support groups. Other studies have also demonstrated that demand creation, when framing PrEP as a way to stay empowered and healthy (rather than as a way to prevent HIV acquisition), could encourage PrEP uptake among young women [25–27]. It is also expected that as PrEP awareness increases, the need for demand creation, and its associated cost, will decline.

Our study did not follow screening procedures that would be done in a MoH setting. Therefore, we excluded screening costs, likely underestimating the cost of PrEP delivery. A recently published PrEP study in young women attending maternal and child health and family planning clinics in Kenya reported a total unit cost (in 2017 USD) of $2.91 for a screening encounter [8]. Future research should evaluate the real-world costs of a comprehensive PrEP program that includes screening, initiation and follow-up visits. To estimate personnel costs, we used the average time a provider spends on each PrEP activity, which does not include the costs of activities not directly related to PrEP delivery such as opening/closing and cleaning the clinic. However, the staff costs would likely be distributed over several staff members, and future research could evaluate costing of an integrated sexual and reproductive health program. Future estimates could also include costs associated with syndromic management of STIs in the MoH scenarios, which we did not consider in this study.

Though we only estimated costs from a provider perspective, costs to clients can be a barrier to PrEP implementation due to inadequate transportation or inability to take time off work or school [23, 24]. In the POWER study, after careful consideration, special accommodations were made for women who were at ongoing risk of HIV acquisition but expressed an inability to return to the facility for a scheduled visit. Specifically, in the costing period, 25 women were given two months of PrEP within the month of PrEP initiation, and during quarterly follow-up visits, one woman was given four months of PrEP and another was given five months of PrEP. This example of a differentiated PrEP delivery, in addition to non-clinic-based service delivery, could reduce costs for PrEP clients and minimize PrEP discontinuation among those indicated for PrEP. Improving access to PrEP through better referral systems can also help reduce costs for PrEP clients, especially during long periods of travel away from the facility of PrEP initiation.

In our study, PrEP visits were scheduled per the Kenyan national guidelines allowing us to estimate costs as they would occur in the MoH scenario. Additionally, though study staff performed the majority of PrEP delivery tasks, some PrEP delivery procedures were performed by facility staff, allowing for their real-world assessment. Specifically, at the private facility (KMET), CrCl, and HBsAg testing and PrEP dispensing were fully conducted by facility staff, while at the public facility (JOOTRH), HIV testing was fully done by facility staff. Costing PrEP delivery procedures as they are performed by facility staff bolsters the usability of our findings to project the local cost of PrEP delivery under MoH implementation.

## Conclusions

In a practical implementation setting, we estimated the cost of PrEP delivery among AGYW in family planning clinics. These data are valuable for informing budget impact and cost-effectiveness analysis to maximize health outcomes for the resources available. In all scenarios, medication was the main contributor of total annual costs, highlighting the need to find ways to lower the price of drugs used in PrEP. Other approaches to minimize costs such as task-shifting, differentiated delivery and prioritization of those at high risk of HIV for PrEP will also need to be evaluated.

## Supporting information

S1 File Spreadsheets and data used for cost calculations. (XLS)
